# Tau in MAPK Activation

**DOI:** 10.3389/fneur.2013.00161

**Published:** 2013-10-17

**Authors:** Chad J. Leugers, Ju Yong Koh, Willis Hong, Gloria Lee

**Affiliations:** ^1^Department of Internal Medicine, University of Iowa Carver College of Medicine, Iowa City, IA, USA

**Keywords:** tau, MAPK activation, phosphorylation, signal transduction, NGF

## Abstract

The nature of “toxic” tau in Alzheimer’s disease (AD) has been unclear. During pathogenesis, the importance of tau oligomerization vs. tau phosphorylation is controversial and the investigation of both remains critical toward defining the “toxicity” of tau. The phosphorylation of tau on serines and/or threonines occurs early in the disease course and altering phosphorylation has been shown to disrupt neuropathogenesis. We have recently reported that in PC12-derived cells, tau had a role in signal transduction processes activated by NGF. By depleting tau, NGF-induced MAPK activation was attenuated and by restoring tau, MAPK activation was restored. Furthermore, the phosphorylation of tau on Thr231 was required for tau to potentiate MAPK activation. Here we report the effects of additional disease-related tau phosphorylation sites and tau isoform on the ability of tau to potentiate MAPK activation. Our findings, which tested three other sites of phosphorylation, showed that phosphorylation at these other sites mainly lessened MAPK activation; none potentiated MAPK activation. In comparing 0N3R tau to the other five brain tau isoforms, most showed a trend toward less MAPK activation, with only 2N4R tau showing significantly less activation. Since MAPK activation has been reported in AD brain and is characteristic of cell proliferation mechanisms, tau phosphorylation that promotes MAPK activation could promote cell cycle activation mechanisms. In neurons, the activation of the cell cycle leads to cell death, suggesting that abnormally phosphorylated tau can be a toxic species. The relationship between tau oligomerization and its ability to potentiate MAPK activation needs to be determined.

## Introduction

The existence of tau pathology occurs in many age-related neurodegenerative diseases that are now termed “tauopathies.” Among these diseases, Alzheimer’s disease (AD) is the most prevalent and it has been suggested that the presence of tau is critical for disease progression (1, 2). Neurodegenerative diseases caused by both missense and intronic mutations in the tau gene have indicated the ability of tau to cause disease [reviewed by Ref. (3–5)]. However, the mechanism by which tau leads to neurodegeneration is unknown. For instance, whether there is a loss of function or a gain of toxic function remains controversial. In considering the role of different tau species during neurodegeneration, hyperphosphorylated tau, and tau filaments have long been investigated. Evidence suggesting that neurofibrillary tangles were not a toxic species came from data indicating that tau-induced behavioral deficits could be improved without changing the tangle burden (6). In fact, neuronal loss did not correlate with neurofibrillary pathology (7). Also, in *Drosophila* and *C. elegans*, tau-induced neurodegeneration occurred in the absence of neurofibrillary tangles (8, 9). Most recently, the investigation of tau oligomers has suggested that they may have an early role in neurodegeneration. Tau oligomers correlate with cellular abnormalities (10–12) and neurodegenerative disease (13–16). However, the molecular mechanism by which tau oligomers cause toxicity has not been clearly demonstrated. In addition, in these studies, the tau oligomers were composed of phosphorylated tau, making it difficult to isolate the effects of oligomerization from those of phosphorylation.

Tau phosphorylation is required for its neurotoxic effects (17, 18) and as tau is hyperphosphorylated early in the disease process, it is not surprising that tau oligomers would be formed from phosphorylated tau. Therefore, in determining if tau oligomers have specific function, one could also first determine the function of abnormally phosphorylated tau, then ask if that tau was in the form of oligomers. Recently, we found that tau has the ability to potentiate NGF-induced MAPK activation and that phosphorylation on Thr231 was critical for the activity (19). Since this activity was seen within 3 h after NGF addition, our data identified a new role for tau in signal transduction processes that take place during neuronal differentiation. At the same time, as Thr231 is phosphorylated early during neurodegeneration (20), it raised the question of whether this new tau activity had a role in neurodegeneration. To further probe the relationship between tau phosphorylation and its ability to potentiate MAPK activation, here we investigate the effects of additional phosphorylated sites, focusing on sites relevant to AD. We also investigate the effects of alternative splicing on the ability of tau to affect MAPK activation.

## Materials and Methods

### Cell culture

PC6-3 cells (21) were cultured on collagen (BD Biosciences) coated dishes using RMPI 1640 medium with 10% horse serum and 5% fetal bovine serum. D5 cells, a stable PC6-3 cell line with stable over-expression of the 0N3R isoform of human tau, and rTau4 cells, a PC6-3 cell line with stable expression of hairpin RNAi targeting endogenous rat tau, were previously described (19). Media for stable cell lines was supplemented with 200 μg/ml G418.

### MAPK reporter assays

MAPK activation was measured by a luciferase reporter assay as described by Leugers and Lee (19). The ability of tau mutants to influence NGF-induced MAPK activation was studied by co-transfecting tau plasmids with the MAPK reporter plasmids. Tau plasmids used were pRc/CMV-0N3R, pRc/CMV-0N3R-S214D, pRc/CMV-0N3R-S404D, pRc/CMV-0N3R-S396D/S404D, pRc/CMV-0N3R-S202D, pRc/CMV-0N3R-S199D/S202D, pRc/CMV-0N4R, pRc/CMV-0N4R-S202D, pRc/CMV-0N4R-S199D/S202D, pRc/CMV-1N3R, pRc/CMV-2N3R, pRc/CMV-1N4R, and pRc/CMV-2N4R. (0N3R, 0N4R, etc., denote tau isoforms where 0N3R contains 352 residues with no amino terminal inserts and three microtubule binding repeats; 2N4R contains 441 residues with two amino terminal inserts and four microtubule binding repeats, etc.). Mutant tau plasmids with phospho-mimicking S to D mutations were constructed using site-directed mutagenesis (Stratagene, Inc.); sequences were confirmed by DNA sequencing.

### Tau detection in cell lines

D5 cells were grown with or without NGF for 30 min and then harvested in RIPA buffer with protease and phosphatase inhibitors (19). After rocking at 4° for 20 min, lysates were centrifuged 20 min. Supernatants were added to an equal volume of 2× Laemmli sample buffer and boiled 5 min. Cell lysate samples were subject to SDS-PAGE and transferred to PVDF membranes. Membranes were probed with anti-phospho-Ser214-tau (Invitrogen, Inc.), PHF1 (22), AT8 (23), Tau5 (24), Tau12 (25), or anti-GAPDH (Chemicon, Inc.). Signal was visualized using ECL (Western Lightning Plus-ECL, Perkin Elmer, Inc.).

## Results and Discussion

To examine MAPK activation, PC6-3 cells, a PC12-derived cell line (21), were treated with NGF. To probe tau function, we used the rTau4 cell line, a PC6-3-derived cell line that expressed a hairpin shRNA that selectively down-regulated the expression of endogenous rat tau without affecting the expression of human tau mediated by transfection (19). In tau-depleted rTau4, NGF-induced MAPK activation was attenuated and the addition of wild-type human tau (0N3R) was able to significantly restore MAPK activity after growth factor treatment (19). Moreover, a phospho-mimicking mutation at Thr231 brought further increases to MAPK activation while a Thr to ala mutation at Thr231 showed a dominant negative effect on MAPK activation (19). This led us to conclude that tau phosphorylation at Thr231 was required for the effect of tau on MAPK signaling. Based on these findings, and the fact that tau can undergo phosphorylation at a number of sites during early brain development (26) or during neurodegeneration (27), we further investigated the effects of tau phosphorylation on MAPK activation.

**Figure 1:**
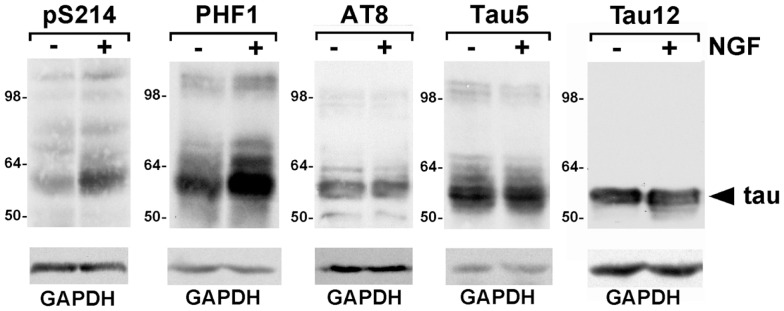
**Phosphorylated tau is expressed in PC6-3 cells**. Serum starved D5 cells were stimulated with 50 ng/ml NGF for 30 min. Cells were harvested as described in Section “Materials and Methods.” Following SDS-PAGE, immunoblotting was performed with antibodies as indicated, total tau being probed by Tau5 and human tau by Tau12. Glyceraldehyde-3-phosphate dehydrogenase (GAPDH) levels are shown as a loading control. Arrowhead indicates human tau; the less abundant rat tau was visualized with Tau5.

To select tau phosphorylation sites to test, we sought sites that were modified in both AD and in the PC6-3 cells. We chose to examine Ser214, Ser396/Ser404, and Ser199/Ser202, all of which are known to be phosphorylated in AD. While tau phosphorylation in NGF-treated PC12 cells has been examined, NGF treatments greater than 24 h were often used and we were interested in earlier time points as our focus was on signal transduction rather than neurite outgrowth. To assess the phosphorylation of both endogenous rat tau and exogenously expressed human tau (0N3R), we utilized the previously described PC6-3-derived cell line D5, that stably expresses human tau (19). In both undifferentiated cells and cells stimulated with NGF, we observed phosphorylation at Ser199/Ser202, detected by AT8, as well as phosphorylation at Ser396/Ser404, detected by PHF1 (Figure 1). NGF treatment was performed for 30 min and no further changes in the level of phosphorylation at these sites were observed for up to 3 h (data not shown). The phosphorylation at Ser396/Ser404 was found to occur in both rat tau and human tau species and the addition of growth factor appeared to slightly increase the level of phosphorylation. Phosphorylation at Ser199/Ser202 was also observed in both rat and human tau and did not appear to change upon NGF addition. In contrast, phosphorylation at Ser214 appeared increased after NGF induction (Figure 1). Our previous data had shown that phosphorylation at Thr231 also increased after NGF induction (19). These data indicate that these tau sites are being phosphorylated in PC6-3 cells.

**Figure 2:**
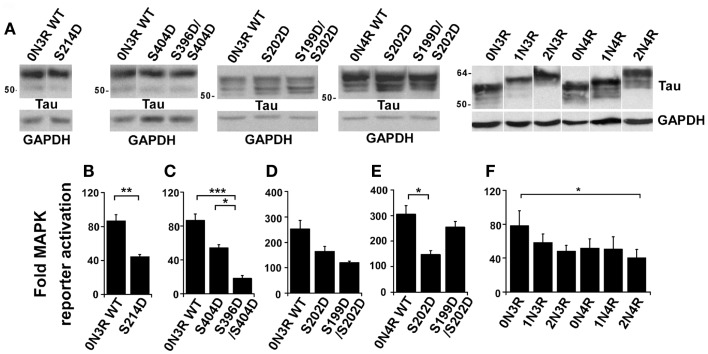
**Tau phosphorylation modulates the effect of tau on NGF-induced MAPK signaling**. rTau4 was transfected with MAPK reporter system plasmids and tau plasmid indicated. Thirty-six hours after transfection, NGF was added 3 h prior to cell harvest. **(A)** To assure equal tau expression in transfections to be assayed in **(B–F)**, lysates from rTau4 cells transfected under identical conditions were probed with Tau13. Glyceraldehyde-3-phosphate dehydrogenase (GAPDH) levels are shown as a loading control. **(B–F)** Fold-MAPK reporter increase was calculated as previously described (19). (*p < 0.05; **p < 0.005; ***p < 0.001). Data shown are mean + SE from three independent experiments; for each experiment, transfections were performed in triplicate for each condition.

To examine MAPK activation, we tested a panel of phospho-mimetic mutations at these tau phosphorylation sites. In advance of measuring MAPK activation, protein produced by each plasmid was visualized by western blotting in order to confirm that equivalent amounts of each protein was being expressed in each experiment (Figure 2A). In this way, differences in MAPK activation would be attributed to protein identity rather than protein quantity. In each experiment, WT tau was expressed in the tau-depleted rTau4 cells as control. When expressing 0N3R human tau with a phospho-mimicking mutation at Ser214 (S214D, Figure 2B), we observed a significant attenuation of MAPK activation relative to WT tau. Next, we tested phosphorylation at Ser404 using S404D and observed a trend of reduction in MAPK signaling (Figure 2C), indicating that phosphorylation at this site might also impair the ability of tau to enhance MAPK signaling. As phosphorylation at Ser404 often occurred in conjunction with phosphorylation at Ser396 (22, 26, 27), we also tested a double mutant, S396D/S404D, and found MAPK signaling significantly impaired relative to both control WT tau and S404D mutant (Figure 2C). In addition, the phospho-mimicking substitution at Ser202 was tested and we observed a trend of decreasing MAPK activation (Figure 2D). The double mutant S199D/S202D was also tested and we found a similar trend (Figure 2D). Together, these findings identified several tau phosphorylation sites where phosphorylation appeared to decrease the ability of 0N3R tau to potentiate MAPK activation. Moreover, these findings indicated that the ability of phospho-Thr231-tau to increase MAPK activation was unique (Table 1).

**Table 1:** **Phospho-mimicking mutations in 0N3R tau, tested for their ability to potentiate MAPK activation**.

	Ability to potentiate MAPK activation
Phospho-Ser214 (S214D)	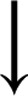
Phospho-Ser202 (S202D)	↓
Phospho-Ser199/Ser202 (S199D/S202D)	↓
Phospho-Ser404 (S404D)	↓
Phospho-Ser396/Ser404 (S396D/S404D)	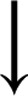
Phospho-Thr231 (T231D)	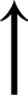

(Bolded larger arrows indicate a statistically significant difference relative to non-phosphorylated tau; non-bolded smaller arrows indicate a trending result.)

Tau mutations that affect the alternative splicing of tau mRNA can result in increased levels of 4R tau and cause neurodegenerative disease [reviewed in Ref. (28, 29)]. To determine if the isoform identity could alter the ability of tau to affect MAPK signaling, we tested different isoforms of tau for their ability to rescue MAPK activation in rTau4. In comparing the abilities of the wild-type 0N3R and 0N4R tau to restore MAPK signaling, while significant differences were not demonstrated, there was a trend showing that 0N4R tau had reduced activity (Figure 2F). In addition, in both 3R and 4R isoforms, we observed a trend of decreased MAPK activation as the N-terminal inserts were added (Figure 2F). A significant difference between the largest and smallest isoforms of tau (2N4R vs. 0N3R tau) was observed (Figure 2F). These observations demonstrated that the effects of tau on MAPK signaling may be modulated by alternative splicing.

Lastly, we compared the effects of the phospho-mimicking substitutions on 0N3R and 0N4R tau. Comparing the effects of S202D on 0N3R and 0N4R, we found that the mutation inhibited MAPK signaling to a larger extent in 0N4R, where a significant decrease occurred (compare Figures 2D,E). However, when the effects of the double mutant S199D/S202D were compared between 0N3R and 0N4R, we found that while 0N3R S199D/S202D resembled 0N3R S202D in its ability to decrease MAPK signaling (Figure 2D), 0N4R S199D/S202D appeared to rescue MAPK signaling, yielding levels similar to wild-type 0N4R tau (Figure 2E). These findings suggest that in 0N4R, while the phosphorylation of tau at Ser202 decreased the ability of tau to potentiate MAPK activation, additional phosphorylation at Ser199 neutralized the effect, returning MAPK activation levels to WT tau levels. These data indicated that the effect of phosphorylation on NGF-induced signaling depended on the tau isoform used.

Our data shows that phosphorylation differentially affected the function of 3R and 4R tau isoforms. This result resembles previous data reported for the interaction between tau and the SH3 domain of Fyn, where we found that phosphorylation differentially affected the equilibrium binding constant of 0N3R and 0N4R tau for the SH3 domain of Fyn (30). For the Fyn SH3 interaction, phosphorylation at Ser199/Ser202 or at Ser396/Ser404 increased the binding of 0N4R to the Fyn SH3 domain whereas phosphorylation at Ser199/Ser202 decreased the binding of 0N3R to the Fyn SH3 (phosphorylation at Ser396/Ser404 did not affect the binding of 0N3R to Fyn SH3). While the details of the effects of phosphorylation are not similar, both the SH3 binding data and the MAPK activation data demonstrate that phosphorylation differentially affected the function of 0N3R and 0N4R tau isoforms. Such findings might help explain why disease could be caused by overexpressing 4R tau relative to 3R tau. In our data, we noted that phosphorylation at Ser199/Ser202 on 4R tau resembled WT tau in its ability to potentiate MAPK signaling whereas the similar modification on 3R tau reduced MAPK activation.

During development, tau phosphorylation changes, with phosphorylation at Ser199, Ser202, Ser214, and Ser404 first increasing, then decreasing while phosphorylation at Thr231 and Ser396 remained unchanged (31). The expression of 4R tau was also up-regulated during development as 0N3R tau was the only tau isoform expressed in fetal brain while the remaining isoforms were expressed in an adult specific manner (32). In our experimental system where NGF was added to initiate neuronal differentiation, phosphorylation could either potentiate or attenuate MAPK activation [Figure 2, Ref. (19)]. Phosphorylation at two sites (Ser214 and Ser396/Ser404) significantly down-regulated activation while that at one site (Thr231) up-regulated activation. Therefore, the exact effects of phosphorylation would depend on the quantity of specific phosphorylated tau forms present. This, in turn, would depend on the rate of phosphorylation and dephosphorylation of tau at specific sites. However, the spatial localization of the various phosphorylated species may also be important. Since we measure MAPK activation in a transfected cell, it is possible that the spatial localization of the tau expressed by transfection may not duplicate that of the endogenous tau. If the ability of tau, expressed by transfection, to affect MAPK activation was dependent on a spatial localization not duplicated by endogenous tau, caution needs to be exercised in the interpretation of our results. Nevertheless, a critical role for tau in MAPK activation was confirmed by our experiments where ERK1/2 activation was examined without the transfection of MAPK reporter plasmids (19).

Our tests have investigated the ability of disease-related tau phosphorylation to affect the ability of tau to upregulate MAPK signaling. Among the sites we have investigated, Thr231 was the site whose phosphorylation occurred earliest during neurodegeneration (33, 34). Phosphorylation at Ser262/Ser356 occurred next, with Ser214 close behind; phosphorylation at Ser199/Ser202 and Ser396/Ser404 accumulated latest during neurodegeneration (33). Therefore, as tau phosphorylation changed during disease progression, tau function would similarly change. Our data suggested that phospho-Thr231-tau would potentiate MAPK activation and since phospho-Thr231 occurred early during the neurodegenerative process (20), one could speculate that MAPK activation would also occur. Data reporting the presence of activated ERK1/2 in pretangle neurons and in Braak stage I–III brains (35) supports the hypothesis that phospho-Thr231-tau may potentiate MAPK activation early in the neurodegenerative process. Then, as tau phosphorylation changed during the neurodegenerative process, the capacity for tau to potentiate MAPK activation would also change. Phosphorylation at Ser262/Ser356 would not affect MAPK activation (19) whereas phosphorylation at Ser214 and Ser396/Ser404 would lead to a decrease in the ability of tau to upregulate MAPK activation (Figure 2). In tauopathies where the level of 4R tau was increased, the isoform change alone might decrease MAPK activation. However, one could also speculate that the phosphorylation of 4R tau, for instance at Ser199/Ser202, could lead to an increase in MAPK activation, relative to that conducted by similarly phosphorylated 3R tau forms. To further investigate the relationship between tau phosphorylation and MAPK activation during neurodegeneration, tauopathy mouse models and/or human post-mortem brain tissue would be probed for phospho-Thr231-tau and activated MAPK. Using immunocytochemistry, if activated MAPK only appeared in the same neurons that were positive for phospho-Thr231 tau, this would suggest that phosphorylation of tau at Thr231 was related to MAPK activation. One would also look for a correlation between activated MAPK and phospho-Ser199/Ser202 in 4R tau isoforms, in addition to a correlation between phospho-Ser214-tau (or phospho-Ser396/Ser404) and a reduction in activated MAPK.

The activation of MAPK can occur in signal transduction pathways where cell proliferation is upregulated [reviewed by Ref. (36, 37)]. Therefore, increasing the activation of MAPK could cause an increase in cell cycle activation. In Drosophila, the ability of tau to cause neurodegeneration was shown to involve cell cycle components (17). Our data supports the hypothesis that tau can potentiate cell cycle mechanisms. In a post-mitotic neuron, the activation of cell division would lead to neurodegeneration [reviewed by Ref. (38)].

It is not known whether tau participates in signal transduction in adult neurons. Gene expression in the tau-depleted mouse was compared to that of WT mouse, using microarray analysis of 8-week-old mice (39). In the tau-depleted mouse, the genes with the highest increase in expression were FosB and c-fos [see Supplemental Data in Ref. (39)]. Since MAPK activation drives fos activation, our data leads us to speculate that the tau-depleted mouse had increased fos expression as a compensatory measure. Since the comparison had used an 8-week-old mouse, it is possible that tau was also necessary for signal transduction in the adult.

While it is clear that phosphorylated tau can form oligomers, we have not determined if the tau that upregulates MAPK activation is a monomer, dimer, trimer, or oligomer. Moreover, the mechanism by which tau affects NGF signaling is under investigation. The ability of proteins to form dimers during signal transduction processes is not unusual and in some cases, dimer formation is linked to phosphorylation. It would be important to determine if tau dimerization or oligomerization occurred in the same manner as disease-related phosphorylation, where dimerization or oligomerization would occur normally during development, then become down-regulated in the adult. If tau oligomers are found during normal development, the toxicity of tau oligomers during neurodegeneration may be related to specific tau oligomer functions that were inappropriate or abnormal for adult neurons.

## Conflict of Interest Statement

The authors declare that the research was conducted in the absence of any commercial or financial relationships that could be construed as a potential conflict of interest.
